# Recent Progress in Probiotic Encapsulation: Techniques, Characterization and Food Industry Prospects

**DOI:** 10.3390/foods15030431

**Published:** 2026-01-24

**Authors:** Zixin Jin, Yi Wang

**Affiliations:** College of Biosystems Engineering and Food Science, Zhejiang University, Hangzhou 310058, China; 22313073@zju.edu.cn

**Keywords:** probiotics, encapsulation, intestinal health

## Abstract

Although probiotics are widely used in the food industry due to their health-promoting effects, their application is often limited by low stability and poor viability under processing, storage, and gastrointestinal conditions. Encapsulation has emerged as a promising strategy to address these issues, offering enhanced protection and controlled release of probiotic strains. This review summarizes recent advances in encapsulation techniques relevant to food applications, including spray drying, freeze drying, coacervation, and liposome formation, as well as novel approaches such as multilayer nanocoatings and dual-core systems. The use of natural biopolymers such as alginate, chitosan, and pectin, along with food-grade synthetic materials, has greatly improved the stability of probiotics in complex food matrices. Furthermore, emerging technologies such as cell-mediated coatings offer improved resistance to gastric acid and oxygen, enhancing probiotic survival through the gastrointestinal tract. These advances contribute to the development of functional foods with better health benefits. However, challenges remain regarding scalability, strain-specific encapsulation efficiency, and regulatory approval. Future research should focus on optimizing food-grade materials, exploring synergistic effects with bioactive compounds, and ensuring consistent performance across food systems.

## 1. Introduction

### 1.1. Probiotics and Their Functional Applications

Probiotics play a critical role in maintaining host health and preventing disease by modulating the gut microbiota, enhancing immune function, and improving metabolic homeostasis [1]. Probiotics primarily consist of members of the genera *Lactobacillus* and *Bifidobacterium*, which are widely used in commercial and therapeutic applications. Additional genera, such as *Saccharomyces*, *Streptococcus*, *Enterococcus*, and *Bacillus*, have been extensively studied and applied as probiotics due to their diverse characteristics.

Probiotics are defined as live microorganisms that, when administered in adequate amounts, confer specific health benefits to the host. Beyond their traditional dominance in fermented dairy products, their application has expanded into diverse matrices including beverages, baked goods, and dietary supplements [2,3,4]. Functionally, probiotics play a dual role: physiologically, they orchestrate gut homeostasis by competitively excluding pathogens, synthesizing short-chain fatty acids, and modulating immune responses to alleviate conditions such as lactose intolerance [5,6]; technologically, they serve as natural biopreservatives that enhance food quality, with their metabolic byproducts—such as organic acids and antimicrobial substances—not only improving flavor profiles but also effectively extending shelf life by slowing spoilage [7]. At present, driven by advancements in encapsulation and processing, the scope of probiotics is shifting towards precision nutrition and personalized functional food interventions.

**Figure 1:**
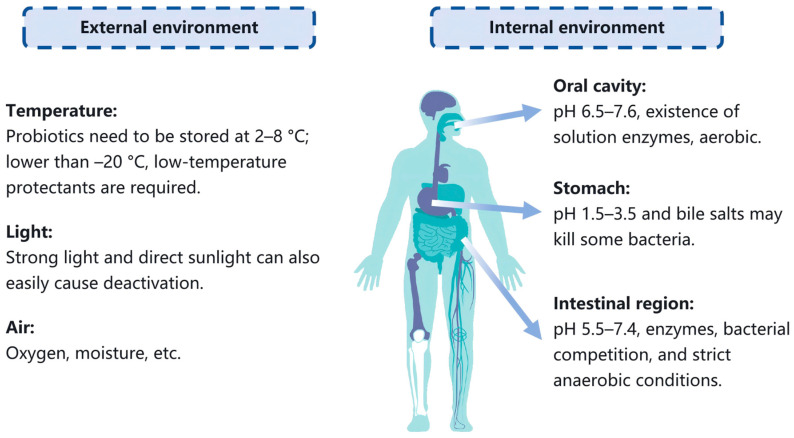
Risk factors for probiotic delivery.

Probiotics are a key area of research in nutrition and health, enhancing the value of food while regulating gastrointestinal microecology. However, harsh conditions such as those associated with gastric acidity, oxygen, and digestive enzymes challenge their survival; for example, exposure to high temperature, humidity, and light during the production and storage phases can inactivate probiotics, reducing their efficacy and shelf life [8]. While probiotics are vital for gastrointestinal health, their activity is diminished in the highly acidic gastric environment. Encapsulation protects probiotics, ensuring their survival through the stomach to the intestine, where they can most effectively exert their beneficial effects [9]. Additionally, oxidative stress due to oxygen exposure can damage cell membranes, decrease metabolic activity, and impair function, particularly for anaerobic strains [10] (Figure 1).

### 1.2. Encapsulation as a Solution for Probiotic Protection

Encapsulation is a sophisticated technique that enhances the stability, activity, and bioavailability of sensitive active substances, such as probiotics, by encapsulating them and ensuring their efficient delivery to targeted sites within the body. Moreover, the controlled release of probiotics can enhance their interaction with the gut microbiota, promoting superior colonization and delivering more substantial health benefits [11]. Research has demonstrated that encapsulation technology can improve the product shelf life, efficacy, and consumer acceptance of probiotics. Consequently, encapsulation technology has emerged as a pivotal strategy in the development of functional foods and dietary supplements [12].

This targeted delivery ensures that more active probiotic cells reach the intestines, maximizing their potential health benefits. Furthermore, in the encapsulation process, additives such as buffers can be incorporated, which further neutralize the acidic environment and provide additional protection for probiotics [13,14]. This innovative approach not only preserves the viability of probiotics but also enhances their ability to colonize and effectively interact with the gut microbiota.

In terms of oxidative damage, encapsulation can significantly reduce the risk of probiotics being exposed to oxygen and reduce such damage by forming a protective barrier; for example, lipid-based encapsulation can create a hydrophobic barrier that not only prevents moisture but also limits oxygen permeability [15]. Additionally, some encapsulation techniques—such as spray drying or freeze drying—enable the incorporation of antioxidants or oxygen scavengers into the encapsulation matrix [16]. These additives can enhance the stability of probiotics by neutralizing free radicals and reducing oxidative stress, thus extending their shelf life and efficacy.

A key advantage of encapsulation is its ability to achieve targeted delivery, precisely delivering probiotics to specific sites in the gastrointestinal tract and thereby enhancing their effects on gut health. Through the selection of appropriate encapsulation materials that respond to specific pH values or enzymatic activities, probiotics can be made to dissolve or degrade at targeted sites within the gastrointestinal tract. This control not only ensures that probiotics reach the intestines intact but also enhances their ability to colonize effectively and exert beneficial effects on the gut microbiota. Another approach for targeted delivery is the use of specific carrier materials to improve adhesion to the intestinal mucosa [17]: through incorporating natural polysaccharides or other bioadhesive agents into the encapsulation matrix, probiotics can adhere more effectively to the intestinal wall, thus increasing their chances of colonization and exerting beneficial activities. As research in this field continues to evolve, the development of more precise encapsulation technologies may lead to more effective probiotic products that meet specific health needs [18].

### 1.3. Scope and Significance of This Review

This review captures the critical paradigm shift in probiotic encapsulation from passive physical barriers to sophisticated, functionalized delivery systems, distinguishing itself from the existing literature by prioritizing emerging bio-interface technologies and structural innovations over conventional techniques. Beyond a comparative assessment of natural and synthetic wall materials regarding their physiological performance, we critically analyze cutting-edge strategies—specifically, multilayer nanocoatings, cell-mediated assembly, and dual-core co-encapsulation—that offer superior biocompatibility and synergistic colonization effects. Crucially, this work bridges the translational gap between laboratory fabrication and industrial application by scrutinizing scalability, cost-efficiency, and regulatory landscapes, ultimately providing a forward-looking roadmap for the development of next-generation, personalized functional foods with precise delivery capabilities.

## 2. Encapsulation Strategies for Probiotic Delivery

**Figure 2:**
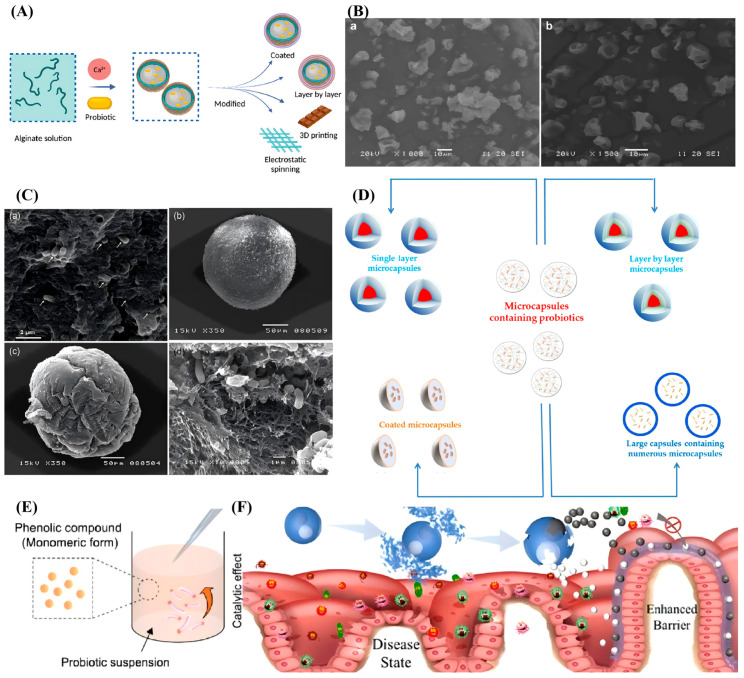
Encapsulation strategies for probiotics. (**A**) Schematic of encapsulation principle [54]. (**B**) SEM images of microcapsules after spray drying; (**a**) scale bar 10 μm, magnification 1000×; (**b**) scale bar 10 μm, magnification 1500× [33]. (**C**) SEM images of (a) casein gel with encapsulated *Lactobacillus paracasei* F19, alginate microcapsules without (b) and with (c) zein shell, and (d) a zein-shelled microcapsule encapsulating *L. acidophilus*; Arrows indicate the encapsulated bacteria [35]. (**D**) Schematic of the multi-layer encapsulation process [28]. (**E**) Mechanism of cell-mediated in situ phenolic nanocoating [29]. (**F**) Microfluidic fabrication of dual-core microcapsules [53].

Encapsulating probiotics poses unique challenges, primarily due to their sensitivity to environmental conditions and the need to ensure their survival during storage and delivery. To address these challenges, various technologies have been developed to enable the effective encapsulation of probiotics. Probiotic encapsulation techniques involve embedding active bacterial populations into protective matrices, isolating them from adverse external conditions [19] (Figure 2A). This significantly enhances the efficacy of probiotics in health interventions and improves the survival rate and stability of microbial populations in the gastrointestinal tract.

To better understand and compare the applications of various encapsulation methods, Table 1 summarizes the characteristics, advantages, and disadvantages of different encapsulation techniques, as well as key parameters that promote the effective encapsulation of probiotics.

### 2.1. Physical Methods

#### 2.1.1. Spray Drying

With ongoing advancements in technology, spray drying has become a mainstream technique for protecting probiotics. The principle involves spraying liquid ingredients as fine droplets (10–150 μm), which are then dried using a hot air stream, typically at temperatures ranging from 150 to 250 °C [20]. D’Amico et al. (2025) developed a composite matrix incorporating inulin, sodium alginate, and maltodextrin for spray drying of Bifidobacterium longum, achieving a high encapsulation efficiency of 97% and a production yield of 90% [21]. This formulation significantly enhanced probiotic survival under simulated gastrointestinal conditions and maintained 93% viability after four weeks of storage at −20 °C. Compared with freeze drying, this technique generally requires less energy and offers higher production efficiency, which is crucial for industrial-scale manufacturing.

Recent advancements have focused on refining related technologies, notably through the incorporation of protective agents into the carrier matrix and the optimization of drying parameters via detailed modeling [31]. Nevertheless, protocols must be tailored to specific probiotic strains, and emerging techniques such as single-droplet technology require further investigation. For instance, Yin et al. (2024) enhanced the survival of *L. rhamnosus* GG (*LGG*; named after Gorbach and Goldin) during spray drying by embedding the bacteria in a solid fat-enriched milk matrix, demonstrating improved probiotic activity when combined with prebiotics [32]. Barbosa et al. (2022) investigated microencapsulation of the next-generation probiotic *Akkermansia muciniphila* DSM 22959 via spray drying, comparing three dairy-based matrices: skim milk, whey protein concentrate, and whey protein isolate [33]. The study determined that 10% SM, processed at inlet/outlet temperatures of 150/65 °C, provided optimal protection, maintaining cell viability at approximately 10^7^ CFU/g after 28 days of aerobic storage at 4 °C with less than a 1 log reduction. Regarding the structural characteristics observed in the SEM analysis (Figure 2B), the optimal microcapsules displayed a spherical-like, deflated, and wrinkled morphology without apparent surface fissures [33]. The authors attributed this shriveled surface texture to the rapid evaporation of water during the atomization and drying process, noting an average particle size of approximately 5.8 μm.

Although spray drying has become the dominant methodology in industrial-scale probiotic encapsulation by virtue of its exceptional cost-efficiency and continuous processing capabilities, its primary limitation lies in the intrinsic thermal stress exerted upon heat-sensitive strains during high-temperature atomization [16]. Therefore, the successful application of this technique is contingent upon the precise engineering of protective matrices to establish a delicate equilibrium between the morphological shrinkage induced by rapid dehydration and the critical preservation of bacterial viability.

#### 2.1.2. Freeze Drying

In practical applications, freeze drying remains the preferred method for preserving probiotics due to its cost-effectiveness and reliability. Kuo et al. (2022) utilized freeze drying as a post-processing step for 3D-printed alginate–gelatin hydrogels, successfully converting them into shelf-stable solid products with water activity reduced to below 0.1 [8]. The study demonstrated that this integrated approach effectively maintained probiotic viability, with *Bifidobacterium lactis* counts exceeding 10^6^ CFU/g after eight weeks of storage at room temperature [8]. However, freeze drying technology also faces several limitations, such as the constrained processing speed. From a technical perspective, the preservation of bacterial strains in industrial applications requires freezing or freeze drying [12]; these processes significantly reduce water activity, which may cause damage to bacterial structures such as cell membranes and intracellular proteins.

Cryoprotectants are widely employed during freeze drying to mitigate cell damage by inhibiting ice crystal formation and stabilizing cell membrane structures, thereby significantly improving the survival rate of probiotics [23]. These protective systems range from conventional sugar- and protein-based cryoprotectants to emerging functionalized polysaccharide matrices. Their effective application typically involves coordinated optimization of bacterial cultivation, cryoprotectant formulation, and freeze drying parameters to achieve maximal protective performance. While freeze drying technology is relatively mature, further research is needed to optimize operational procedures, select appropriate cryoprotectants, and define parameters tailored to specific probiotics.

The primary difference between spray drying and freeze drying lies in the thermodynamic mechanisms and the mode of action of the protectants. Spray drying relies on rapid dehydration at high temperatures, forming an amorphous matrix including protectants such as trehalose, resulting in smooth particle shrinkage. In contrast, freeze drying inhibits the damage caused by ice crystal formation at low temperatures, with protectants stabilizing the cell membrane via glass transition; as such, its effectiveness is dependent on the solid concentration and osmotic regulation [34]. The scanning electron microscope (SEM) results shown in Figure 2C provide intuitive evidence, visually validating the correlation between the encapsulation morphology of cells and the protectant mechanisms under the two drying modes.

Nevertheless, the demanding conditions needed to maintain vitrification pose substantial barriers to industrial application. In contrast to the rapid, continuous operation of spray drying, lyophilization is a slow, energy-intensive batch process [35]. In addition, prolonged cycles escalate the associated operational costs to nearly ten-fold that of spray drying, posing a formidable barrier for cost-sensitive formulations [36]. Moreover, industrial upscaling is complicated by the difficulty of maintaining uniform heat transfer in large chambers, when compared with laboratory settings. Thus, although freeze drying is essential for preserving high-value, heat-sensitive strains, scalability and efficiency issues limit its feasibility for mass-market functional foods.

### 2.2. Chemical Methods

#### 2.2.1. Coacervation

The complex coacervation method primarily involves dispersing the core substance in a polymer solution with opposite charges. Through adjusting factors such as temperature and pH, the wall material precipitates on the surface of the core substance due to charge interactions and reduced solubility, thus forming microcapsules. This method has been widely studied in food systems, particularly for protein- and polysaccharide-based coacervation systems. Proteins and polysaccharides with opposite charges can undergo electrostatic interactions to form microcapsules [22]. Narmin et al. (2024) utilized soybean isolate protein (SPI) as the matrix and combined gelation with other methods to construct SPI-based probiotic microcapsules with high structural density and crosslinking [22]. The addition of pectin (PEC) to SPI significantly improved the survival rate of SPI during gastrointestinal digestion and storage, providing better protection. Ma et al. (2024) investigated the use of corn zein and chitosan (CS) in a coacervation reaction to construct bilayer microcapsules with a hydrophobic core and a hydrophilic shell, serving as dual-functional factor delivery carriers [37].

Compared with traditional single-component coacervation methods, such an approach offers better environmental adaptability and protective effects for probiotics. Zhao et al. (2020) demonstrated the effectiveness of this method in encapsulating *L. reuteri* TMW1.656, identifying optimal conditions (pH 6.0, GE/Cas ratio of 2.0) [38]. They showed that the water–water emulsion maintained bacterial viability during spray drying, simulated digestion, heat stress, and ambient storage, outperforming the gelatin/gum arabic system. While coacervation effectively mitigates gastrointestinal stressors through precise protein–polysaccharide interactions, as demonstrated for the robust SPI–pectin and zein–chitosan frameworks, its broad application necessitates rigorous control over physicochemical parameters such as pH and temperature to maintain the delicate charge balance for optimal shell formation.

#### 2.2.2. Endogenous Emulsion

Endogenous emulsions are formed through a type of emulsification method, the key principle of which is to mix insoluble calcium salt particles and alginate to form a uniform and stable solution, which is then injected into an oil phase [25]. Under the action of the active agent, an emulsion is formed. By passing H^+^ through the surfactant layer (O/W interface), acidic substances are added to decrease the reaction pH, thus leading to the loss of calcium ions from the insoluble calcium salt. Then, alginate is incorporated to form a gel microcapsule ball inside the wrapped liquid drop [26]. The endogenous emulsion method overcomes the clustering phenomenon of microcapsules, making the particle size of microcapsules easy to control, and enables the formation of microcapsules, which are smaller and present a more uniform, wide distribution range, better sphericity, and a smoother surface.

The endogenous emulsion method has been studied for the encapsulation of certain probiotics due to the resulting small particle size and high uniformity of the prepared microcapsules. Bansal et al. (2016) encapsulated oxaliplatin (L-OHP) within folic acid-conjugated liposomes, which were subsequently entrapped in alginate beads and further coated with Eudragit S-100 to enhance their targeted delivery to colon tumors [39]. Peng et al. (2025) demonstrated the superiority of endogenous emulsification over traditional extrusion for encapsulating *L. piracies* ProSci-92 [40]. While the extrusion method was hindered by nozzle clogging and irregular particle formation due to the high viscosity of the sodium alginate–soy protein isolate composite, the endogenous emulsification strategy enabled precise morphological control. By optimizing the process parameters, they achieved a remarkable encapsulation efficiency of 92.17%, significantly outperforming the extrusion approach. Mechanistically, this enhanced performance was attributed to the amphiphilic and emulsifying properties of the soy protein isolate (SPI), which stabilized the ionic gel network of sodium alginate (SA), thus forming a dense, defect-free microstructure. While research on endogenous emulsification has focused on enhancing static stability, recent advancements in Pickering emulsions have shifted towards intelligent, stimuli-responsive systems to address the challenge of bacterial recovery. Jiang et al. (2025) engineered a thermo-responsive Pickering emulsion using hydrophobic silica-armored PNIPAM microgels [41]. This system functions as a dynamic microcompartment: at 37 °C, it forms a stable water-in-oil (w/o) emulsion that supports anaerobic cultivation, achieving a 6.3-fold increase in biomass compared with traditional methods. Uniquely, lowering the temperature to 25 °C triggers a spontaneous phase inversion to an oil-in-water (o/w) state, allowing for the rapid, non-destructive separation of the bacterial suspension with high viability (23.1%), overcoming the retrieval bottleneck inherent to conventional emulsion carriers.

However, the industrial translation of endogenous emulsification faces intrinsic constraints regarding downstream processing and formulation precision. Unlike aqueous-based extrusion, this technique relies on a continuous oil phase, necessitating rigorous harvesting and washing steps to eliminate residual solvents and surfactants that could otherwise compromise the sensory profile and safety of food products [42]. Furthermore, the mechanism hinges on precise stoichiometric balance between the insoluble calcium salt and the acid trigger; deviations from optimal ratios often lead to unpredictable gelation kinetics, resulting in either incomplete crosslinking or heterogeneous internal matrices that weaken the protective barrier.

### 2.3. Multi-Layer Embedding

Due to the complex cellular environment of the human gastrointestinal tract, different wall materials can be effectively processed through multi-layer embedding. As shown in Figure 2D, multi-layer embedding provides an additional layer of protection, effectively isolating the probiotics from contact with the external environment [28]. This helps to mitigate the adverse effects of extreme pH environments such as those in the gastrointestinal tract on probiotics and delays their release in the colon, thus enhancing their stability in designated environments. Moreover, multi-layer embedding enables different release mechanisms to be designed at various levels, allowing probiotics to be released specifically at the inflammation site, and more precise control over the release rate of probiotics can be achieved through the selection and design of different materials [43]. This controlled release mechanism helps to optimize the location and effect of probiotics in the gut, improving the benefits conferred to the host. Compared with traditional single- or double-layer embedding, multi-layer embedding also allows functional layers to be added (e.g., incorporating nanomaterials or bioactive ingredients), thus providing additional health benefits to probiotic products and improving their overall efficacy.

Han et al. (2024) developed a method to encapsulate probiotics in curcumin-loaded liposomes, followed by further coating with polymers using a layer-by-layer technique [43]. Their results indicated that multi-layer encapsulation at the nanoscale increases the size of probiotics and improves their survival rate under simulated gastrointestinal conditions, highlighting the potential of multi-layer embedding to enhance the efficacy of probiotics in treating colitis-related diseases. The mild strengthening and coating processes prevent severe damage to the sensitive probiotics during processing, while the synthesized multi-layer macroparticles protect the probiotics against gastric acid and bile salts. Moreover, the solubility of the outer shell and the stable inner porous structure under intestinal conditions allow for the gradual release of the encapsulated probiotics in targeted environments [43].

Multi-layer embedding represents a paradigm shift from passive encapsulation to programmable delivery systems within the food industry. Its primary advantage lies in a hierarchical architecture that decouples functional roles, allowing specific layers to independently withstand processing stressors (e.g., heat) or physiological triggers (e.g., acidity) [44]. This structure enables the precise engineering of functional foods with targeted colonic release properties. However, this sophistication imposes a complexity penalty regarding industrial scalability, as the requisite multi-step assembly protocols often conflict with high-throughput manufacturing. Furthermore, the cumulative increase in particle size risks exceeding sensory detection thresholds and introducing undesirable granularity into smooth food matrices.

### 2.4. Cell-Mediated Nanocoatings

Inspired by the metabolic processes of living cells and cell-mediated chemical reactions, researchers have attempted to develop biomimetic materials with higher biocompatibility. Fundamentally, this technique is distinguished from passive physical deposition by exploiting the active physiological responses of the probiotics [45]. It involves the direct in situ formation of a protective shell on individual cell surfaces, triggered by the secretion of endogenous catalysts (e.g., manganese ions) or specific metabolic reactions when the bacteria are exposed to precursor solutions [46]. Specifically, through cell-mediated biointerfacial assembly, active substances—including natural active materials, effective health ingredients, and therapeutic drugs—can be incorporated into the surface of probiotics [47]. This not only enhances the functions of probiotics but also creates more possibilities for disease prevention and the development of new health products.

Centurion et al. (2022) demonstrated that the cell-mediated catalytic process enables the in situ formation of phenolic polymer nanocoatings on individual probiotics, enhancing gastric tolerance (increased by 1.4×) and intestinal adhesion (increased by 1.6×), while also imparting antioxidant activity (Figure 2E) [29]. Therefore, cell-mediated nanocoating is considered an important approach for achieving innovation in probiotic encapsulation technology. Phenolic compounds are widely utilized in such processes, as their catechol- or gallol-rich structures facilitate the rapid self-assembly of dense metal–phenolic network (MPN) barriers via coordination, while simultaneously providing intrinsic antioxidant activity to scavenge interfacial reactive oxygen species [48]. Lim et al. (2025) constructed a multifunctional nanocoating on *L. plantarum* utilizing green tea residue-derived polyphenols and cellulose nanocrystals (CNCs) [49]. Driven by cell-mediated manganese secretion, a polymeric phenolic shield was catalytically assembled on the bacterial surface, which subsequently anchored the CNCs. This green nanocoating not only provides robust antioxidative protection and resistance to gastrointestinal stresses but also significantly enhanced intestinal mucoadhesion via the integrated CNCs, as evidenced by a 2.5-fold increase in mucin adsorption capacity and a peak adhesion rate of 56.8% to intestinal epithelial cells.

In contrast to conventional encapsulation techniques, nanocoating establishes an ultra-thin, uniform nanoshell directly on individual bacterial surfaces. This single-cell encapsulation strategy ensures equipotent protection for every bacterium, resulting in superior structural homogeneity [50]. Moreover, due to its negligible thickness, the nanocoating preserves the original rheological behavior and sensory attributes of the final food or pharmaceutical matrix.

### 2.5. Collaborative Inclusion of Two Bacteria

In addition to novel encapsulation technologies, the use of traditional methods to simultaneously embed multiple substances—especially combinations of different probiotics—represents an innovative and promising approach [51,52]. This comprehensive encapsulation strategy not only broadens the scope of applications but also demonstrates superior performance compared with conventional single-substance encapsulation methods. Through integrating multiple active ingredients within a single delivery system, this approach unlocks more possibilities for synergistic interactions, enhanced stability, and improved therapeutic outcomes.

A prominent example of this strategy is the work by Zhao et al. (2020), who developed dual-core microcapsules using electrostatically driven microfluidics to encapsulate *Lactobacillus* and *Bacillus subtilis* in separate compartments [53]. Figure 2F shows that this design ensures that each probiotic strain is shielded from environmental stressors while preserving its viability and functionality. The microcapsules exhibited remarkable acid resistance, maintaining the activity of both bacterial strains during gastrointestinal transit. Furthermore, the encapsulation system created an anaerobic microenvironment that supported the proliferation of the probiotics and promoted lactic acid fermentation, without cross-interference between the two strains. These distinctive features make dual-core microcapsules a promising candidate for addressing metabolic syndrome and related disorders, in the context of which the synergistic action of multiple probiotics offers multifaceted health benefits [52].

The simultaneous encapsulation of multiple substances or probiotics not only enhances their individual effects but also fosters synergistic interactions, thereby amplifying their overall efficacy. These synergistic effects can lead to improved bioavailability, enhanced functional performance, and greater therapeutic potential compared with single-substance systems.

## 3. Encapsulation Materials for Probiotics

The selection of wall materials is of paramount importance in probiotic microencapsulation, as it directly dictates the protective capacity against processing stressors, storage instability, and gastrointestinal barriers. An optimal matrix not only ensures the structural integrity of the microcapsules but also facilitates controlled release and targeted colonization within the host gut.

### 3.1. Natural Polymers

#### 3.1.1. Polysaccharides

Natural materials are increasingly being utilized as wall materials for the encapsulation of probiotics due to their biocompatibility, biodegradability, and functional properties. Among these materials, polysaccharides such as alginate, chitosan, and pectin are particularly favored due to their ability to form stable gels and provide effective protection against environmental stressors. These natural wall materials not only protect probiotics from adverse factors such as heat and moisture but also facilitate targeted release in the intestines, thereby maximizing the associated health benefits.

1. Alginate

As a biocompatible, inexpensive, and non-toxic material, alginate has been widely used in probiotic encapsulation—an essential approach to enhancing the stability, viability, and controlled delivery of probiotics, particularly within the harsh conditions of the gastrointestinal tract [55]. Natural polysaccharides, including sodium alginate, carboxymethyl cellulose (CMC), and chitosan, have been widely explored due to their ability to form hydrogels that effectively protect probiotics from environmental stressors [56]. These polysaccharides not only provide an ideal environment for encapsulation but also contribute to controlled release dynamics, improving the effectiveness of probiotics.

In particular, sodium alginate has been extensively studied due to its ability to form gels that protect probiotics during encapsulation and transit through the stomach. Li et al. (2023) developed a composite system incorporating South jujube fruit peel polysaccharide (CP) or its stable water-in-oil emulsion as a filler into calcium alginate microgels to encapsulate *L. casei* [57]. They found that CP and its emulsion reduced gel viscosity while significantly enhancing antioxidant activity, maintaining probiotic viability (>7.0 log CFU/g) under simulated gastrointestinal conditions and 20-day storage. This material not only demonstrates a means for the high-value utilization of natural waste but also opens a new pathway for the development of low-cost, environmentally friendly probiotic delivery systems. At the same time to ensure the best encapsulation efficiency, it is often necessary to design a large number of specific parameters relating to the encapsulation process of sodium alginate, where different parameters may lead to completely different encapsulation efficiency. For this purpose, Thinkohkaew et al. (2024) employed regression methods to calculate the relevant parameters and optimized encapsulation outcomes through use of the calculated extreme values, achieving a notable improvement [58].

**Figure 3:**
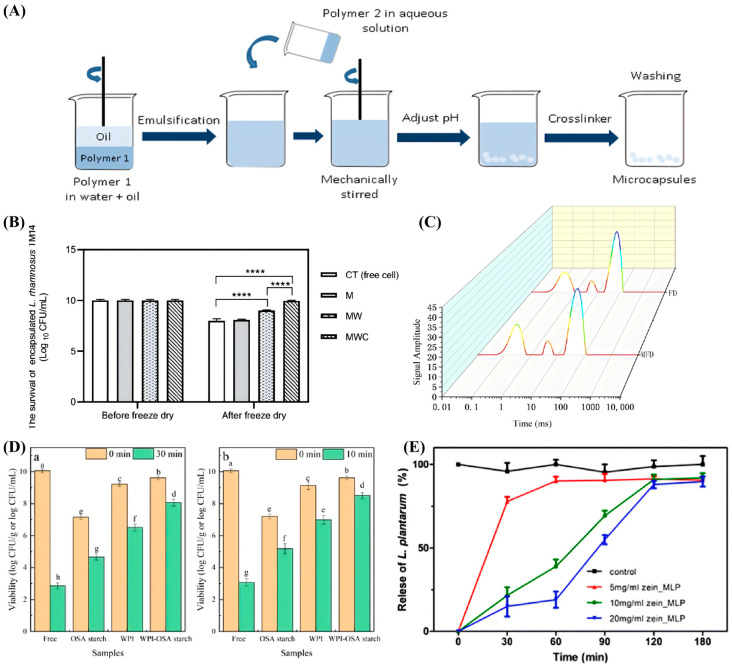
The protective effects of probiotic encapsulation materials. (**A**) The complex coacervation method based on polysaccharides [59]. (**B**) Effect of composite cryoprotectants on probiotic survival during freeze drying [60]. (**C**) Moisture distribution status under different drying methods, **** *p* < 0.0001 [80]. (**D**) Viability of free and microencapsulated *L. acidophilus* after thermal treatment of (**a**) 65 °C/30 min and (**b**) 75 °C/10 min [89]. (**E**) Zein encapsulation protects probiotics against SGF [96]. The letters a-h indicated a significant difference.

To further overcome the mechanical limitations of single-network hydrogels, researchers have leveraged the process of electrostatic complexation between oppositely charged biopolymers to reinforce the structural integrity of the encapsulation matrix. For instance, Santos et al. (2018) highlighted the use of interpolymeric complexes formed between whey proteins and polysaccharides as a superior encapsulation strategy (Figure 3A) [59]. Through manipulation of the pH and ionic strength, these biopolymers can form a dense, structurally reinforced network via electrostatic interactions, effectively overcoming the porosity and instability often associated with single-component gels [59]. This reinforced matrix acts as a robust physical barrier for probiotics, such as *Lactobacillus* species, significantly enhancing their resistance to gastric acidity and ensuring high viability during processing and storage.

The addition of further protective agents, such as maltodextrin, has also been shown to enhance probiotic survival. For example, in a calcium alginate–maltodextrin hydrogel system, *L. rhamnosus* was encapsulated using electrospray and freeze drying techniques. Maltodextrin significantly reduced the surface porosity and pitting, resulting in better stability and improved viability of the encapsulated probiotics. This method effectively extended the survival of probiotics in the gastrointestinal tract, especially when compared with the use of alginate alone, which had limited protective effects on *L. casei* [56]. According to Poopan et al. (2025), the results directly indicated that, compared with free cells (CT group), the cell survival rates of probiotics encapsulated with maltodextrin (M group); with maltodextrin and whey protein (MW group); and with maltodextrin, whey protein, and corn flour (MWC group) were significantly improved (Figure 3B) [60].

Through combining materials such as sodium alginate, CMC, chitosan, and maltodextrin, researchers have been able to create more stable, efficient, and protective environments for probiotics, enabling their survival under harsh digestive conditions and improving their efficacy in promoting gut health. Rao et al. (2025) demonstrated that calcium pectin beads (CPB) and sodium alginate-pectin-whey beads (SPW), as composite materials, achieved different results in encapsulating probiotics [61]. Specifically, microcapsules including SPW as a wall material exhibited a more solid and complete appearance, indicating higher cross-linking density and better resistance to high temperatures and gastrointestinal environments [61].

2. Pectin

Pectin, a natural polysaccharide primarily extracted from citrus fruits and apples, is widely recognized for its gel-forming capabilities and excellent biocompatibility, making it an ideal candidate for probiotic encapsulation. Under the presence of calcium ions, pectin forms hydrogels that provide a robust protective barrier for probiotics. Wang et al. (2024) reported a microencapsulation process that involved embedding bacteria and spores in a double-layered microcapsule using microfluidic technology, which demonstrated high viability under various stress conditions, significantly improving survival rates and reducing disease activity in mice with acute colitis [62].

Using a similar approach, Sun et al. (2024) developed a hydrogel microbead system incorporating montmorillonite into a pectin–alginate matrix for the encapsulation of *L. kefiri* [63]. This system enhanced the probiotic’s stability during gastrointestinal delivery and storage, with the incorporated montmorillonite facilitating the formation of a layered cross-linked network on the surface of the microbeads, thus minimizing the loss of viability in gastric environments and promoting controlled, slow release alongside effective colonization in the gut. Furthermore, the viability of *L. kefiri* during storage was maintained with minimal reductions. Melanie et al. (2024) reported the equivalent diameters of hydrated microbeads—determined via stereo microscopy—to range from 2.40 ± 0.01 mm to 3.02 ± 0.11 mm [64]. Although larger beads are generally theoretically associated with better protection, the authors found that the pectin-rich formulations—which showed the smallest diameters in their study—successfully achieved high encapsulation yields (87–96%) and stability under simulated gastrointestinal conditions.

Furthermore, pectin’s ability to dissolve in the presence of gastrointestinal enzymes ensures controlled release in the intestines, allowing the probiotics to optimally exert their beneficial health effects [62]. Beyond its encapsulation properties, pectin’s soluble fiber content is advantageous for gut health, adding a synergistic element to probiotic formulations.

3. Chitosan

Chitosan has gained increasing attention in various fields due to its distinct properties and advantages. In the context of probiotic encapsulation, chitosan forms a protective matrix that shields probiotics from environmental stressors such as heat, moisture, and acidity, thereby significantly enhancing their viability during storage and transportation [61]. Additionally, chitosan’s positively charged nature facilitates interactions with negatively charged bacterial membranes, promoting improved adhesion and colonization within the gastrointestinal tract [65].

Chitosan can also be combined with other materials (e.g., alginate or pectin) to create composite structures with enhanced stability and that enable controlled release of probiotics in the gastrointestinal environment. For instance, Mehrana et al. (2024) encapsulated *L. plantarum* using a chitosan–casein composite via a layer-by-layer strategy involving calcium phosphate cross-linking [66]. This approach significantly improved the survival and antioxidant activity of the probiotic under simulated digestive conditions. The encapsulated *L. plantarum* demonstrated superior detoxification of Aflatoxin M1 compared with the free bacteria, underscoring the effectiveness of this biocompatible encapsulation method in enhancing probiotic functionality. Finally, given its natural origin and multifunctional properties, chitosan stands out as an exceptional material for developing effective probiotic formulations in functional foods and dietary supplements.

4. Modified starch

Modified starch is widely utilized in the encapsulation of probiotics due to its versatile properties and ability to protect sensitive microorganisms. As a natural polysaccharide derived from starch, modified starch undergoes chemical or physical modifications to enhance its functionality, making it an ideal material for encapsulation [66,67]. Modified starch forms a protective coating around probiotic cells, shielding them from environmental factors such as heat, moisture, and gastric acidity. This protective layer significantly enhances the stability and viability of probiotics during processing, storage, and passage through the gastrointestinal tract [68].

In a separate study, Wang et al. (2022) prepared propionylated potato starch (PPS) with varying degrees of substitution, using potato starch as the raw material [69]. They studied its embedding performance for *LGG*, finding that PPS with a substitution degree of 1.54 exhibited the highest encapsulation rate. Moreover, this PPS demonstrated strong acid resistance and stability under low-temperature storage, providing a novel method for probiotic encapsulation.

Additionally, modified starch possesses excellent film-forming and emulsifying properties, facilitating the creation of stable microcapsules that ensure the controlled release of probiotics in the intestines. Zhu et al. (2023) investigated the encapsulation of *L. plantarum* using porous starch (PS) through mild gelatinization, demonstrating that the amylose/amylopectin ratio influences the pore size and encapsulation efficiency [70]. PS can accommodate *L. plantarum* cells within its pore structure, depending on the amylose content. Furthermore, the size and number of pores in PS significantly affect the number of live cells encapsulated in the process. According to Zhu et al. (2023), when compared with PS prepared using traditional enzymatic methods, these innovative PS formulations exhibited superior encapsulation efficiency and higher probiotic stability under mild gelatinization conditions [70].

5. Cellulose

As a natural polysaccharide derived from plant cell walls, cellulose is renowned for its mechanical strength, stability, and biocompatibility. In the context of probiotic encapsulation, cellulose can be modified to form various derivatives, such as microcrystalline cellulose or carboxymethyl cellulose, which enhance its functionality [71,72]. For example, Li et al. (2023) pioneered the use of Millettia speciosa Champ cellulose (MSCC) and carboxymethylcellulose (MSCCMC) to create a 3D-network hydrogel for the delivery of *L. paracasei* BY2 [73]. This approach demonstrated high encapsulation efficiency (up to 88.91%) and controlled release (up to 92.86%), both of which were correlated with the concentration of MSCCMC.

Moreover, the excellent film-forming properties of cellulose facilitate the controlled release of probiotics in the gastrointestinal tract, ensuring that a higher number of viable cells reach the intestines to optimally exert their health benefits [74]. Additionally, as a dietary fiber, cellulose contributes to gut health, making it a synergistic component in probiotic formulations. Jayani et al. (2020) developed bacterial cellulose nanofibers (BCNFs) via electrospinning with polyvinyl alcohol (PVA) to encapsulate *L. acidophilus*, demonstrating successful immobilization and sustained viability of the probiotic bacteria [75]. The BCNFs exhibited suitable mechanical and thermal properties for food applications, further confirming their potential as a viable probiotic delivery system.

6. Gum Arabic

Gum arabic—a natural gum obtained from the acacia tree—is widely used for the encapsulation of probiotics due to its excellent emulsifying and stabilizing properties. However, Zhao et al. (2018) mapped the associated probiotic inventory curve under long-term storage conditions, revealing that low temperature and low humidity remain the most favorable conditions for the long-term preservation of bacterial strains [76].

Chen et al. (2023) co-encapsulated diacylglycerol (DAG) oil and probiotics using gelatin–gum arabic complex coacervates, demonstrating enhanced probiotic resistance to environmental stresses, including freeze drying, heat treatment, and simulated digestive conditions [77]. The microcapsules exhibited superior cell viability and membrane stability, highlighting the potential of DAG oil co-encapsulation for improving probiotic survival under adverse conditions.

Additionally, gum arabic promotes the controlled release of probiotics in the gastrointestinal tract, improving survival rates and facilitating their colonization in the intestines. In parallel, Ma et al. (2023) employed electrohydrodynamic techniques—including electrospinning and electrospraying—to encapsulate *L. plantarum* KLDS 1.0328 using synthetic and natural biopolymers [78]. They reported enhanced probiotic survival under simulated gastrointestinal stress and thermal processing conditions, particularly with polyvinyl alcohol/gum arabic (PVOH/GA) nanofibers. Gum arabic’s prebiotic effects also support the growth of beneficial gut bacteria, further enhancing overall gut health. The biocompatibility and biodegradability of gum arabic make it an ideal choice for developing functional food products and dietary supplements aimed at efficiently delivering live probiotics.

#### 3.1.2. Proteins

1. Gelatin

Gelatin is a widely utilized encapsulation material for probiotics, which is renowned for its excellent biocompatibility and biodegradability. As a thermoresponsive polymer, it undergoes phase transitions in response to temperature fluctuations, allowing it to form stable matrices that effectively protect probiotics against environmental stressors during both processing and storage [79]. To enhance the encapsulation efficiency, Chen et al. (2023) utilized transglutaminase (TGase) to crosslink gelatin (G) and sodium hexametaphosphate (SHMP) as wall materials for the microencapsulation of *L. plantarum* [80]. They further compared the efficacy of microwave freeze drying (MFD) versus traditional freeze drying (FD) [80]. The proton density images presented in Figure 3C reveal the moisture distribution status under different drying methods; in particular, the FD samples exhibited areas with high signal intensity, indicating the presence of residual bound water that is difficult to remove. In contrast, the MFD samples displayed a uniform blue color with lower proton density, demonstrating that the microwave treatment facilitated more efficient removal of deep-seated moisture compared with FD. This superior dehydration efficiency, combined with the dense TGase-crosslinked G/SHMP network, resulted in microcapsules characterized by a survival rate of 86.41% and significantly improved viability under simulated gastrointestinal conditions and long-term storage. Notably, when combined with microwave vacuum freeze drying, the optimal transglutaminase (TGase) concentration of 9 U/g Gelatin strengthened the hydrogel’s structural integrity and protective effects, indicating a promising strategy for probiotic delivery.

One of the notable characteristics of gelatin is its ability to dissolve in warm water and form gels upon cooling, which facilitates the controlled encapsulation of probiotics [81]. This gelation process not only provides a protective barrier but also enables the targeted release of probiotics in the gastrointestinal tract [82]. By manipulating the composition and processing conditions, gelatin capsules can be engineered to dissolve at specific temperatures or pH levels, ensuring that a greater number of viable probiotics reach their intended destination in the intestines. In addition, gelatin demonstrates film-forming properties that enhance its adhesive qualities, promoting better adhesion to the intestinal mucosa. This improved adhesion supports the colonization of probiotics, enabling them to exert their beneficial effects more effectively.

2. Whey protein

Whey protein is increasingly employed in the encapsulation of probiotics due to its exceptional functional properties and nutritional benefits. As a byproduct of cheese production, whey protein is rich in essential amino acids and bioactive compounds, making it a valuable ingredient in probiotic formulations [83,84]. This protective barrier not only enhances probiotic stability during storage and transit but also facilitates controlled release in the gastrointestinal tract [85]. Moreover, whey protein exhibits emulsifying properties that contribute to the stability of probiotic suspensions [86].

Etchepare et al. (2020) demonstrated that encapsulating *L. acidophilus* in calcium alginate particles with multilayer coatings—particularly with whey protein—significantly improved probiotic survival under simulated gastrointestinal conditions, thermal treatment, and storage [87], underscoring the efficacy of multilayer microencapsulation in enhancing probiotic viability and stability. The combination of probiotic bacteria with whey protein can also enhance the overall health benefits of the product, as whey protein has been shown to support immune function and muscle health. Ngamekaue et al. (2024) utilized whey protein isolate (WPI) and coconut oil (CO) emulsions to encapsulate *Limosi L. reuteri* KUB-AC5 via spray drying [88]. Their results demonstrated enhanced probiotic survival and reduced cell envelope injury, particularly with 4% CO-WPI emulsions, which exhibited superior viability during simulated gastrointestinal digestion.

Leveraging the superior physicochemical properties of proteins, Liu et al. (2023) utilized Whey Protein Isolate (WPI) as the primary encapsulation matrix for *Lactiplantibacillus plantarum* LIP-1 [89]. The significance of WPI lies in its amphiphilic nature and excellent film-forming capacity, facilitating the formation of a dense, protective network through electrostatic interactions with cationic polymers such as chitosan. This reinforced WPI-based structure significantly enhanced the thermal stability of the probiotics [89]; for instance, under heat stress at 70 °C for 30 s, the microencapsulated cells maintained a survival rate of 84.63%, which was notably higher than that of the free cells (60.36%), thus effectively addressing the challenge of viability loss during thermal processing (Figure 3D).

Overall, the use of whey protein in probiotic encapsulation not only provides an effective means of delivering viable microorganisms but also contributes additional nutritional value to functional foods and dietary supplements [90].

3. Zein

Corn protein, particularly zein, is increasingly used for probiotic encapsulation due to its unique properties and versatility [91,92]. Derived from corn, zein is a hydrophobic protein that forms a stable matrix, making it highly effective in protecting probiotics from environmental stressors such as heat, moisture, and acidity [93]. This protective barrier significantly enhances the viability and stability of probiotics during storage and transport, ensuring that a higher percentage of viable cells reaches the gastrointestinal tract. Additionally, zein’s film-forming capabilities facilitate controlled release, enabling probiotics to be delivered effectively to the intestines, where they can exert their health benefits [94,95].

Cheng et al. (2024) investigated the encapsulation of *Bacillus subtilis* using zein and soluble soybean polysaccharide (SSPS) nanoparticles, formed via antisolvent precipitation [93]. The formed nanoparticles demonstrated improved probiotic viability under simulated gastrointestinal digestion, pasteurization, and storage conditions, with in vivo experiments confirming an increase in beneficial gut bacteria in rats. This study emphasized the effectiveness of zein-based formulations in enhancing probiotic stability and functionality. Distinct from conventional single-component zein matrices, which may lack sufficient structural density to fully prevent acid penetration, Zeng et al. (2024) constructed a robust double-layer microencapsulation system to overcome these limitations [96]. Employing zein as the hydrophobic inner core and a sodium alginate/pectin complex as the hydrophilic outer shell, they successfully encapsulated *L. plantarum* via electrostatic deposition. The functional significance of this zein-based composite architecture is vividly illustrated in Figure 3E, which depicts the viability of the probiotics during in vitro simulated gastrointestinal digestion [96]. The results demonstrate that, while free bacteria were severely compromised by gastric fluid, the Zein/Sodium Alginate/Pectin (Z/SA/P) microcapsules provided exceptional protection, maintaining a high viable count of 6.91 log CFU/mL after the complete digestive process. This confirms that the zein core, when reinforced by a polysaccharide shell, creates a superior physical barrier against environmental stresses.

Finally, similar to zein, quinoa protein has also emerged as a promising candidate with broad application prospects. Fan et al. (2025) demonstrated that nanocoating *L. fermentum* RC4 with a complex of hyaluronic acid and quinoa protein significantly enhanced probiotic stability, viability, and functional activity [97]. Mechanistically, this improvement was attributed to the modulation of structural interactions and gene expression [97].

#### 3.1.3. Lipids

1. Animal fats

Animal fats—although less commonly used in probiotic encapsulation compared with other materials—offer distinct advantages due to their natural lipid composition and protective properties. Fats such as lard or tallow can be utilized to create fat-based microcapsules, providing protection against environmental stressors such as heat, moisture, and gastric acid, thus enhancing the viability of probiotics during storage and transport. The hydrophobicity of animal fats serves as an effective barrier against oxygen, thereby reducing oxidative damage in probiotics and improving their overall stability and shelf life [98]. Silva et al. (2022) evaluated modified anhydrous milk fat as an encapsulating material for *L. acidophilus* LA3, demonstrating high probiotic viability after 60 days of storage [99]. They also showed that encapsulation with this material provided better physical stability and dispersibility at lower temperatures, underscoring its potential for use in various food matrices.

2. Waxes

Waxes (e.g., beeswax, carnauba wax, and paraffin wax) have been employed for probiotic encapsulation due to their unique properties, offering a range of potential applications. Their solid structure at room temperature makes them particularly suitable for creating microcapsules that can withstand environmental stressors while maintaining the integrity of the probiotics [100]. In addition, waxes can be utilized in controlled release systems as they are gradually broken down in the digestive system, allowing probiotics to be released slowly in the intestines, thus improving their survival rate. Waxes are often combined with other encapsulation materials, such as polysaccharides or proteins, to create composite systems that further enhance the protective capabilities of the probiotic coating. The use of waxes in encapsulation offers a non-toxic, natural, and effective barrier, ensuring that the probiotics reach their intended target.

3. Phospholipids

Phospholipids (e.g., lecithin) are widely used in probiotic encapsulation due to their amphiphilic nature and ability to form lipid bilayers, thus enabling several important applications. Phospholipids can form liposomes—that is, small spherical vesicles—that encapsulate probiotics, providing a protective barrier against environmental stressors such as oxygen, moisture, and gastric acid [101]. This protection enhances the stability and viability of probiotics during both storage and gastrointestinal transit.

Zhang et al. (2023) investigated the co-encapsulation of *L. plantarum* WCFS1 and algal oil via complex coacervation with gelatin and gum arabic [102]. They highlighted the crucial role of soy lecithin (a phospholipid) in enhancing the stability and lipolysis of the microcapsules; in particular, the addition of soy lecithin significantly improved the viability of probiotics during freeze drying and storage, as well as the release and digestion of algal oil. These results demonstrated the potential for effective delivery of both probiotics and omega-3 fatty acids to the intestine.

Phospholipids also excel in promoting controlled release, ensuring that probiotics are delivered to specific sites in the intestines. Their lipid bilayer structure mimics that of natural cell membranes, improving the bioavailability of the encapsulated probiotics. Additionally, phospholipids are characterized by their biocompatibility and biodegradability, making them ideal for use in both food and pharmaceutical applications [102]. Furthermore, they can be combined with other encapsulation materials to enhance the protective properties and functionality of the delivery system.

#### 3.1.4. Pickering Emulsions

Emulsion encapsulation technology is used to establish protective microenvironments for probiotics against gastrointestinal conditions by constructing physical barriers at oil–water (O/W, W/O) or water–water (W/W) interfaces [103]. Among these, water-in-water (W/W) Pickering emulsions have emerged as advanced delivery vehicles due to the biocompatibility of their fully aqueous components and tunable interfaces. For instance, hydroxypropyl methylcellulose/maltodextrin biphasic systems stabilized with cellulose nanocrystals (CNC) enable probiotic encapsulation within the dispersed phase. Subsequent acid-triggered Ca^2+^ release from CaCO_3_ in the dispersed phase induces in situ crosslinking with interfacial sodium alginate, forming an “egg-box”-structured dense barrier. This approach was shown to improve probiotic viability during simulated gastrointestinal digestion by three orders of magnitude (7.56 × 10^7^ CFU/mL), significantly outperforming conventional emulsion systems [104].

Du et al. (2025) stabilized high internal phase Pickering emulsions with a 90% oil fraction using AGLs at a 1% concentration, breaking conventional emulsifier dosage limits [105]. This system achieved gastrointestinal-targeted delivery of probiotics, elevating the survival of *L. reuteri* to over twice that of free bacteria. Crucially, the approach involves preparing agar–lauric acid composite particles (AGLs) via one-step alkaline ball milling, thus substituting traditional chemical modification with green physical processing to significantly enhance the hydrophobicity of natural agar, thereby resolving its emulsification constraints due to excessive hydrophilicity.

Overall, future emulsion systems are expected to evolve towards higher efficiency and functional diversity, making them a promising research direction in the field. Further studies may focus on optimizing component ratios and interfacial structures to reduce microbial antagonism within the emulsion, thereby improving the stability and viability of the encapsulated probiotics. In addition, developing tailored emulsion carriers—especially those characterized by resistance to high-temperature and shear conditions—will be key to meeting the diverse encapsulation needs of different probiotics during processing and storage.

### 3.2. Synthetic Polymers

Synthetic materials are increasingly utilized in probiotic encapsulation due to their tailored properties and ability to provide effective protection. Materials such as poly(lactic acid) (PLA), poly(lactic-co-glycolic) acid (PLGA), and polyethylene glycol (PEG) are among the most popular choices for creating robust microcapsules. These synthetic polymers can be engineered to achieve specific release profiles, thereby allowing for controlled delivery of probiotics in the gastrointestinal tract. In addition, synthetic materials can be formulated to incorporate various additives, such as prebiotics or antioxidants, which further enhance the overall efficacy of probiotic products [106]. The versatility and customization options of synthetic materials make them particularly suitable for developing advanced probiotic formulations in functional foods and dietary supplements. This ensures that the live microorganisms reach their target site in optimal condition, thereby maximizing their potential to exert health benefits.

#### 3.2.1. Poly(Lactic-co-Glycolic) Acid

Poly(lactic-co-glycolic) acid (PLGA) is a widely utilized synthetic polymer for probiotic encapsulation, owing to its favorable biodegradability and tunable release properties [107,108]. Comprising lactic acid and glycolic acid, PLGA can be engineered to achieve specific degradation rates, enabling the controlled release of probiotics over time; this feature is particularly advantageous for ensuring that a higher number of viable probiotic cells reach the gastrointestinal tract, where they can exert their health benefits. Ajalloueian et al. (2022) developed a multilayer electrospun construct to enhance the delivery of *LGG* by encapsulating the probiotic within pullulan nanofibers sandwiched between protective PLGA layers [108]. This innovative design significantly improved the viability and storage potential of *LGG*, demonstrating superior performance in both in vitro and in vivo studies, with notable colonization in the intestine [108]. PLGA provides a robust protective barrier against environmental stressors such as heat and moisture, significantly enhancing the stability of probiotics during storage and transport [109]. Furthermore, its biocompatibility and safety profile make it well-suited for food and pharmaceutical applications. The ability to incorporate various additives, such as prebiotics or protective agents, further improves the efficacy of PLGA-based probiotic formulations [107].

#### 3.2.2. Polyvinyl Alcohol

Polyvinyl alcohol (PVA) is increasingly utilized in probiotic encapsulation due to its exceptional film-forming properties and biocompatibility. As a synthetic polymer, PVA is water-soluble, enabling the formation of stable microcapsules that effectively protect probiotics from environmental stressors such as heat, moisture, and acidity [110]. Ma et al. (2024) pioneered the use of electrospinning to fabricate PVA/fucoidan (FUC) composite nanofibers, which were used to encapsulate the probiotic *L. plantarum* 69-2 (LP 69-2) along with four polyphenols [111]. Wei et al. (2021) successfully performed encapsulation using PVA and polyvinyl alcohol/silk fibroin (PVA/SF) nanofibers via electrospinning [112] and demonstrated that PVA significantly enhanced the survival rate under simulated gastric conditions, thus presenting a novel strategy for protecting probiotics in challenging environments. Additionally, PVA can be modified to adjust its release profile, enabling the controlled delivery of probiotics to specific areas of the digestive system [111]. Its non-toxic nature and capacity to form hydrogels further indicate PVA as an ideal material for use in various functional food and dietary supplement applications [113].

#### 3.2.3. Polyethylene Glycol

Polyethylene glycol (PEG) is a versatile synthetic polymer that is employed in probiotic encapsulation due to its excellent solubility, biocompatibility, and ability to form stable hydrogels [114]. PEG effectively protects probiotics against environmental stressors such as heat, moisture, and acidity, thereby enhancing their viability during storage and transit. Its hydrophilic nature facilitates the incorporation of probiotics into various formulations, while enabling controlled release in the gastrointestinal tract [115]. Through adjusting the molecular weight of PEG, manufacturers can fine-tune the release profile of probiotics to optimize their delivery and effectiveness [116]. Additionally, PEG can be combined with other materials to create composite systems that enhance the stability and functionality of probiotic formulations. The non-toxic and biodegradable properties of PEG make it an attractive option for developing functional foods and dietary supplements, ensuring that the live microorganisms reach their target site in optimal condition to exert their health benefits.

Demonstrating the practical application of such composite systems, Bayat et al. (2024) recently utilized a polycaprolactone–polyethylene glycol–polycaprolactone triblock copolymer to micro-coat *Bifidobacterium lactis* for fortification in Iranian white cheese [114]. Capitalizing on the amphiphilic properties of the PEG segment, this copolymer formed a robust protective barrier that significantly mitigated the impacts of gastric acidity and bile salts. Experimental data revealed that while free bacteria experienced rapid viability loss, the PEG–copolymer-coated probiotics maintained a high survival count of 10^6^ CFU/g for up to 45 days under simulated intestinal conditions.

## 4. Evaluation of Encapsulation Performance

### 4.1. Physical and Structural Characterization

**Figure 4:**
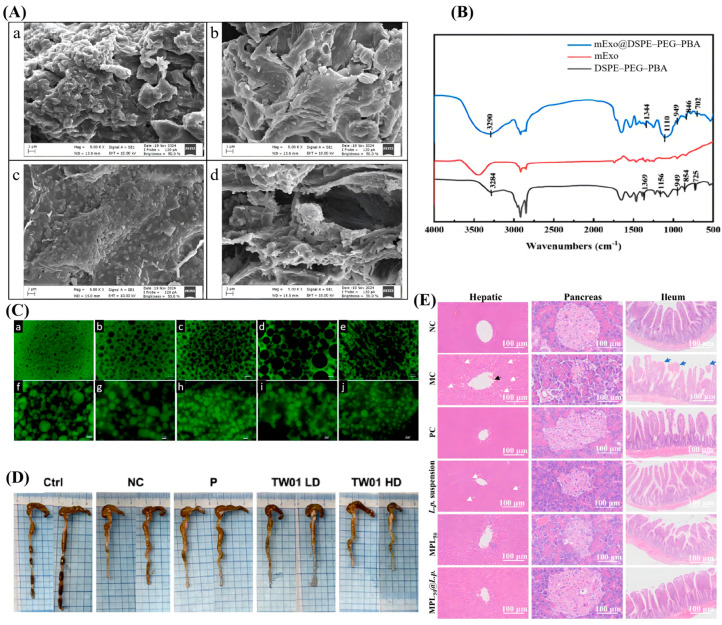
Validation of the probiotic encapsulation system through multidimensional characterization. (**A**) The SEM images: (**a**) Carob flour, (**b**) sterilized carob flour, (**c**) carob flour–*L. rhamnosus* capsules, and (**d**) carob flour–*L. paracasei* capsules [117]. (**B**) Chemical composition analysis using FTIR [118]. (**C**) The fluorescence microscope image of Gelatin/Dextran W/W emulsions consisting of 10 wt% Gelatin with (**a**) 1.5 wt%, (**b**) 3.0 wt%, (**c**) 4.5 wt%, (**d**) 6.0 wt%, (**e**) 7.5 wt%, (**f**) 9.0 wt%, (**g**) 10.5 wt%, (**h**) 12.0 wt%, (**i**) 13.5 wt%, and (**j**) 15.0 wt% Dextran, respectively [119]. (**D**) Macroscopic observation of the colon in a mouse model of colitis [145]. (**E**) H&E-stained tissue sections illustrating wound healing in mice (Black arrows: enlarged hepatic central veins; white arrows: disordered hepatocytes and congested hepatic sinusoids; blue arrows: ileal mucosal erosion/villi rupture) [147].

To investigate the structure and properties of functional materials, researchers have utilized a variety of complementary techniques. Scanning electron microscopy (SEM) is commonly employed to visualize surface features and particle sizes [108]. This technique is especially useful for examining probiotics before and after processes such as drying, providing valuable insights into structural changes. For example, Tülek et al. (2025) employed SEM to characterize the microstructure of microcapsules loaded with *L. rhamnosus* and *Limosilactobacillus fermentum* [117]. The micrographs revealed that the microcapsules maintained a continuous and intact polymeric matrix, despite the morphological roughness induced by the drying process. Crucially, the SEM analysis confirmed the absence of deep fissures or exposed bacterial cells on the surface, providing visual evidence that the wall materials formed a robust physical barrier (Figure 4A). This structural integrity is fundamental to the enhanced viability observed in subsequent storage and gastrointestinal digestion tests, directly linking the microscopic morphology to the macroscopic protective performance of the encapsulation system [117].

When combined with attenuated total reflection Fourier transform infrared spectroscopy (ATR-FTIR), SEM allows for both physical dimension analysis and molecular structure characterization through the observation of chemical bond vibrations. In the study of Hao et al. (2025), FTIR provided critical chemical evidence for the successful assembly of the encapsulation layer through the detection of specific chemical bonds (e.g., B-O bonds and benzene rings) characteristic of the coating material on the probiotic surface (Figure 4B) [118].

In probiotic research, fluorescence microscopy is a valuable tool for assessing the viability and distribution of cells. This technique involves treating samples with fluorescent dyes under controlled conditions, followed by the removal of excess dye and examining the samples under a microscope to measure fluorescence intensity. This intensity correlates with cell activity and membrane integrity [62]. Fluorescence microscopy enables the visualization of encapsulation efficiency, making it possible to study the controlled release behavior of probiotics in a simulated environment such as the gut or stomach. Wu et al. (2025) employed fluorescence microscopy to visualize the phase morphology of water-in-water emulsions, proving the occurrence of phase inversion where the dispersed phase transforms into a continuous phase, as driven by changes in the dextran concentration (Figure 4C) [119].

### 4.2. Physicochemical Stability Mechanisms

#### 4.2.1. Thermodynamic State and Ice Crystal Morphology

The deleterious impacts of moisture on probiotic survival extend beyond simple hydration, being intrinsically linked to the thermodynamic state of the encapsulation matrix. Biologically, residual water functions as a low-molecular-weight plasticizer that increases the free volume within the polymer matrix [120], facilitating the transition of the encapsulating material from a rigid glassy state to a mobile rubbery state, thereby reducing the viscosity of the local environment [121]. Consequently, this enhanced molecular mobility eliminates the kinetic barrier against diffusion, allowing atmospheric oxygen and reactive species to permeate the matrix and exponentially accelerate oxidative degradation [122]. Thus, the glass transition temperature (T_g_)—as the boundary line between the rubbery state and the glassy state—effectively reflects the important factors that affect the stability of probiotic encapsulation materials [123]. For instance, Li et al. (2023) encapsulated *L. plantarum* LP90 using Maillard reaction conjugates of whey protein and carboxymethylated dextran [124], and found that extending the reaction time raised the degree of grafting, leading to the formation of a more rigid molecular network and significantly elevating the T_g_ to 156.31 °C. This high-T_g_ matrix effectively preserved the glassy state during processing, achieving a freeze drying survival rate of 87.37% and superior storage stability.

Beyond molecular kinetics, the physical state of water fundamentally governs the mechanical integrity of cells during freezing processes [125]. The rate of nucleation dictates the morphology of ice crystals: while rapid cooling favors the formation of small, benign crystals, slow freezing regimes induce the growth of large, needle-like extracellular ice crystals that can mechanically puncture the cell envelope. Furthermore, high water activity during storage can trigger ice recrystallization, where smaller crystals merge into larger ones, exacerbating physical damage and confirming the pivotal role of water control in maintaining the viability of probiotics [126]. SEM is also instrumental in visualizing the microstructural footprint of ice crystals (e.g., pore dimensions). Through correlating these morphological observations with probiotic survival rates before and after lyophilization, the specific impacts of ice crystal size on bacterial viability can be rigorously evaluated [127].

#### 4.2.2. Oxidative Stability and Evaluation Strategies

Oxygen exposure represents a critical lethal factor for many probiotic strains, particularly obligate anaerobes and microaerophiles [128]. The presence of atmospheric oxygen leads to the accumulation of reactive oxygen species, which induces severe oxidative stress and results in lipid peroxidation of the cell membrane, metabolic inactivation, and irreversible functional impairment [129]. To mitigate such damage, the encapsulation materials function as a protective shield by forming a physical barrier that effectively isolates the bacterial core from the external environment. Through the utilization of wall materials with low oxygen permeability or incorporating antioxidants within the matrix, the diffusion of oxygen can be significantly reduced, thereby maintaining a stable micro-anaerobic environment for the cells [130]. To verify the efficacy of these oxygen barriers, researchers typically employ a combination of biological and physicochemical characterization techniques. In vitro viability assays under oxidative stress conditions—such as exposure to hydrogen peroxide—provide primary quantitative indicators of protective capacity. By exposing microcapsules to defined concentrations of H_2_O_2_, researchers can simulate accelerated oxidative environments; in this context, a higher survival rate relative to free cells serves as the primary indicator of barrier integrity. Furthermore, mechanistic analysis is often deepened through the use of ROS-sensitive fluorescent probes (e.g., DCFH-DA) combined with fluorescence microscopy. This approach allows for the in situ visualization of intracellular oxidative levels, providing direct cytological evidence that the encapsulation matrix effectively blocks the transmission of oxygen or chemically scavenges permeating radicals before cellular damage occurs [131].

### 4.3. Biological Testing

Simulating the internal (e.g., gastrointestinal) environment is essential for evaluating the performance of encapsulated probiotics or other substances. To simulate the intestinal conditions, trypsin is mixed with a 0.5% sodium chloride and 4.5% bile salt solution to a concentration of 1 g/L, and the pH is adjusted to 7.4. This solution is then filtered through a 0.2 μm filter. These simulated environments facilitate in vitro microbial experiments, which are critical for establishing the groundwork for subsequent in vivo studies. Thinkohkaew et al. (2024) simulated both intestinal and gastric conditions, with their experimental results showing that microencapsulation significantly enhanced the survival rate of probiotics in the simulated environment, meeting the required bacterial cell count for probiotic efficacy [58].

Furthermore, considering that the viability of probiotics often declines when exposed to various external stressors, such as heat, light, acidity, and elevated oxygen levels, it is crucial to assess whether microencapsulation can enhance their resistance to these environmental challenges. Chen et al. (2024) investigated the survival rates of probiotics under different stress conditions, including heat, hydrogen peroxide, osmotic pressure, and freeze drying stress, providing valuable insights into the protective effects of microencapsulation on probiotic viability [132].

In addition to cell viability, the encapsulation efficiency (EE%) serves as another critical parameter for evaluating the effectiveness of encapsulation. To determine the EE%, a sample containing encapsulated cells is first suspended in a buffer solution, followed by serial dilution and plating onto agar plates. After incubation, the number of colony-forming units (CFUs) is counted. The material containing the encapsulated cells is then washed with buffer, and the supernatant is separated via centrifugation. The diluted supernatant is subsequently plated onto an agar plate and, after another round of incubation, CFUs are counted again. The encapsulation efficiency is calculated by comparing the total number of cells to the number of unencapsulated cells. This provides a quantitative measure of the effectiveness of the encapsulation process. This method facilitates a comprehensive assessment of encapsulation, ensuring that cells are not only viable but also securely encapsulated. The final calculation method is as follows:

(1) $$EE\left(\%\right)=\frac{N}{N_{0}}\times 100$$

where *N* is the number of viable cells (log CFU/mL) released from the microcapsules, and *N*_0_ is the number of viable cells (log CFU/mL) of the strain used for the microcapsules. Table 2 summarizes the encapsulation efficiency and survival rates of probiotics associated with various wall materials and encapsulation methodologies.

Considering the requirements for practical applications, thermal and storage stability are critical parameters. Thermal stability is assessed by dispersing the sample in a buffer solution, subjecting it to heat treatment under various conditions, and halting the reaction with rapid cooling; activity or stability is then evaluated using fluorescent reagents or other methods. Storage stability is evaluated by storing the sample at different temperatures for a set period, with regular sampling and monitoring of activity or stability using fluorescent reagents or suitable techniques [144].

From a production and application standpoint, an extended shelf life is a prerequisite for the successful industrialization and practical use of probiotic encapsulation technologies. Liu et al. (2023) investigated the storage capacity of various formulations and demonstrated that microcapsules coated with chitosan exhibited a higher activity (0.2 Log CFU/mL) after 60 days of storage at 4 °C, with an increased coating thickness correlating with enhanced stability [94].

### 4.4. In Vivo Studies

Although in vitro findings highlight the promise of probiotic delivery systems, the in vivo environment presents distinct physiological challenges such as enzymatic activity, immune responses, and microbiota competition. Consequently, rigorous validation in animal models is essential to confirm the translational value of these systems. This assessment typically focuses on critical parameters including intestinal colonization efficiency, therapeutic efficacy in disease models, and biodistribution safety.

To specifically illustrate how encapsulation enhances therapeutic outcomes in a pathological context, Luo et al. (2025) constructed a model of acute ulcerative colitis by having C57BL/6 mice drink a 3% DSS solution for 7 days [145]. For the experiment, the mice were randomly divided into a normal group, a model group, and drug treatment groups (low/high doses) [145]. The results showed that the mice in the model group exhibited significant colonic shortening (a macroscopic indicator of inflammation severity) and destruction of the mucosal structure, while the colonic length was significantly restored and the histopathological score was improved in the drug treatment groups, indicating that the drug could effectively alleviate intestinal inflammation (Figure 4D). In addition to therapeutic efficacy in disease models, evaluating colonization stability and host safety is equally critical. For instance, Ajalloueian et al. (2022) assessed the in vivo performance of *LGG*-loaded nanofibers using a streptomycin-treated Sprague Dawley rat model [108]. Following a single oral administration of 10^9^ CFU, they monitored fecal bacterial counts and physiological indicators over 7 days. Comparative analysis revealed that the encapsulated formulation significantly prolonged *LGG* retention in the gut compared with the administration of free bacteria. Furthermore, the absence of adverse symptoms such as significant weight loss, diarrhea, or rectal bleeding confirmed the biocompatibility and safety of the delivery system [108].

To corroborate the macroscopic therapeutic efficacy observed in disease models, rigorous microscopic and molecular assessments are indispensable for elucidating the underlying protective mechanisms [146]. Histological analysis (e.g., H&E staining) allows for direct visualization of tissue repair and structural integrity, while immunological quantification (e.g., ELISA) enables verification of the modulation of specific inflammatory pathways. For instance, in a recent study utilizing streptozotocin-induced T2DM rats, Liu et al. (2025) employed H&E staining to assess the structural integrity of key metabolic organs following the administration of a Pueraria lobata-derived colon-targeted delivery system (MPL_50_@L.p.) [147]. The histological analysis revealed that the diabetic control group suffered from severe multi-organ damage characterized by hepatic sinusoid congestion, pancreatic islet atrophy with cytoplasmic vacuolation, and ileal mucosal erosion [147]; in contrast, the encapsulated *L. paracasei* treatment effectively reversed these pathological alterations by restoring the orderly arrangement of hepatic lobules, preserving the distinct boundaries of pancreatic islets and repairing the intestinal villus architecture (Figure 4E). These morphological findings provide direct evidence that the targeted delivery strategy not only modulates metabolic parameters but also actively mitigates diabetes-associated tissue deterioration.

Furthermore, beyond therapeutic outcomes, determining the precise biodistribution and colonization kinetics within the gastrointestinal tract is fundamental to validating a delivery system’s targeted performance and host safety. Unlike simple fecal counts, which only reflect elimination, segmental intestinal analysis provides high-resolution data on bacterial retention in specific gut regions. Exemplifying this strategy, Ajalloueian et al. (2022) implemented a comprehensive colonization assay in healthy Sprague Dawley rats [108]. Administering a defined dose of 10^7^ CFU and collecting luminal contents from the jejunum, ileum, cecum, and colon at distinct intervals (24 and 72 h), they successfully mapped the specific biodistribution of the bacteria, confirming that the nanofiber encapsulation significantly enhanced retention across the gastrointestinal tract compared with free bacteria [108].

In addition, Zhou et al. (2024) combined in vivo experiments with fluorescence imaging to intuitively show that a porous microgel provides supplementary protection for *Akkermansia muciniphila*, thus minimizing the consumption of probiotics before reaching the inflamed colon [148]. In addition, no fluorescence signals were observed in major organs such as the heart, liver, spleen, lungs, and kidneys, indicating that the relevant substances were mainly metabolized in the gastrointestinal tract, and their diffusion to other major organs after oral administration can be ignored. By combining in vivo and in vitro approaches, researchers can gain a more holistic understanding of the efficacy and safety of probiotic delivery systems, validating their potential for further clinical applications.

Fluorescence imaging serves as a pivotal characterization tool in probiotic encapsulation research, primarily to visualize the core–shell architecture and verify the integrity of the protective coating, which is a prerequisite for ensuring subsequent in vivo gastrointestinal survival and colonization. Specifically, in a study focused on colitis therapy, Wang et al. (2024) utilized fluorescence microscopy to validate the successful assembly of chitosan-coated pectin microcapsules [62]. By observing the distinct fluorescence signals, the researchers confirmed the formation of a dense “core–shell” gel network, where the chitosan layer effectively coated the pectin core containing *Clostridium butyricum* spores. This verified structural integrity provided the mechanistic basis for the enhanced therapeutic efficacy observed in the DSS-induced acute colitis mouse model. The dense coating minimized the premature leakage of probiotics in the upper gastrointestinal tract, thereby enabling the microcapsules to significantly reduce the DAI and maintain colon length in the treatment group compared with free bacteria controls [62].

## 5. Challenges of Probiotic Encapsulation

### 5.1. Cost of Encapsulation Techniques

The financial burden associated with probiotic encapsulation remains a significant challenge, especially for small-scale producers, researchers, or manufacturers aiming to bring products to a broader market.

First, the cost of the encapsulation materials plays a crucial role. While small-scale production in laboratories often allows for the use of more expensive and effective materials, commercialization depends on the use of cheaper encapsulation materials. For example, natural polymers such as alginate, chitosan, and pectin are widely used due to their biocompatibility, but their extraction and purification processes can be quite costly. Producing high-purity chitosan from crustacean shells requires extensive deproteinization and desalting steps, which can increase the production cost by 20–30% compared with synthetic alternatives like PLGA [149]. Meanwhile, synthetic polymers offering customized release properties are typically derived from expensive raw materials and involve complex synthesis pathways. Therefore, developing cheaper and more easily produced encapsulation materials should also be a focus for researchers.

Second, the specific costs associated with drying methods also play an important role. While spray drying is energy-efficient for large-scale production, it requires specialized equipment (e.g., high-pressure nozzles and cyclone separators) and protective additives (e.g., maltodextrin) to reduce heat-induced cell damage; notably, these additives can account for 40–50% of the total encapsulation cost [150]. Moreover, such complex equipment is often unaffordable for small-scale manufacturers. Meanwhile, although freeze drying offers high reliability, it is energy-intensive. Due to extended processing times and the need for cryoprotectants, its operational cost can reach 10 times higher than that of spray drying. Innovative methods, such as multi-layer encapsulation and cell-mediated nanocoating, which involve advanced technologies such as microfluidics and electrostatic deposition, further escalate research and production costs; for example, producing dual-core microcapsules through electrostatic driving of microfluidics requires precision equipment, which can increase the cost by 35–50% compared with single-layer systems [150].

Third, developing encapsulation schemes for specific strains requires a significant amount of experimentation to determine optimal parameters, further driving up research and development costs; for example, optimizing the protective matrix for oxygen-sensitive strains such as *Bifidobacterium* requires repeated testing with antioxidants or oxygen scavengers, increasing R&D costs by 15–20% [151]. These excessive costs may prevent probiotic encapsulation technology from benefiting a wider population. Therefore, the development of more affordable and scalable probiotic encapsulation products that can be mass-produced is equally deserving of attention from researchers.

### 5.2. Scale-Up Issues for Commercial Production

1. Process consistency

Despite the effective performance of laboratory-scale encapsulation techniques—such as gelation or liposome formation—in small-batch production, batch-to-batch variation becomes a significant challenge in large-scale industrial production; for example, when preparing microcapsules using alginate–chitosan composites, the expansion of the reactor volume may reduce the mixing efficiency, leading to uneven particle size distribution of the microcapsules (the standard deviation of particle size in laboratory-scale production is typically ±5 μm, whereas that in industrial-grade production may reach ±20 μm) [58]. This non-uniformity directly affects the release kinetics of probiotics, potentially resulting in premature release of some batches of products in the gastric environment, while other batches may fail to effectively colonize the intestines due to excessive encapsulation, which is clearly unacceptable for consumers.

To address this issue, researchers have attempted to optimize process parameters such as the stirring speed, temperature gradient, and material feeding sequence, integrated with online monitoring technologies to adjust the production process in real time. As an example, the use of computational fluid dynamics (CFD) to simulate the fluid dynamics within the reactor can help to predict mixing dead zones and optimize equipment design, thereby controlling the coefficient of variation for particle size to within 5% [152]. Additionally, the introduction of artificial intelligence algorithms to analyze historical production data allows for the dynamic adjustment of process parameters, further improving process stability.

2. Equipment Limitations

Many advanced encapsulation technologies rely heavily on precision equipment—such as electrostatic microfluidic systems or high-pressure spray dryers—which are often not inherently designed for high-throughput production. This limitation becomes particularly evident when scaling these processes to meet industrial demands. Taking spray drying as an example, although its single-batch treatment capacity can reach hundreds of liters, significant challenges arise during scale-up, including an inability to precisely control critical parameters such as droplet size and the uniformity of the hot air distribution [153]. In industrial settings, spray dryers often exhibit considerable variability in performance; for instance, the hot air temperature fluctuation in industrial spray dryers can reach ±10 °C, resulting in uneven thermal conditions within the drying chamber. Such inconsistencies lead to overheating in certain areas (with temperatures potentially exceeding 80 °C), causing thermal inactivation of sensitive probiotics, while insufficient temperatures in other regions compromise drying efficiency and product quality [154]. Empirical data further underscores the severity of this issue, revealing that these temperature deviations can reduce the survival rate of probiotics by as much as 30–50%. These technical limitations highlight the urgent need for innovations in both equipment design and process optimization to ensure scalability without compromising product integrity.

### 5.3. Variability Among Probiotic Strains

The encapsulation process for probiotics is highly strain-dependent, with significant variability observed across different strains. These differences are primarily driven by the unique physiological characteristics of each strain, including their oxygen sensitivity, acid resistance, and specific metabolic requirements [155]. Certain strains of *Lactobacillus*, such as *LGG*, exhibit strong acid resistance but remain vulnerable to mechanical damage caused by ice crystal formation during freeze drying. Studies have shown that this mechanical stress can lead to a 30–40% loss of biological activity [35].

This inherent variability among probiotic strains necessitates the development of tailored encapsulation solutions to address the specific needs of each strain; for example, to enhance the survival of oxygen-sensitive *Bifidobacterium*, antioxidants such as glutathione or vitamin C can be incorporated into the encapsulation matrix. Additionally, advanced techniques such as multi-layer embedding can be employed to create a physical barrier that effectively isolates the bacteria from exposure to oxygen [156]. Such customized approaches can be expected to not only improve the resilience of probiotics during processing but also to ensure better preservation of their functional properties, ultimately enhancing the overall quality and efficacy of the final product.

## 6. Prospects and Suggestions for Encapsulation of Probiotics

### 6.1. Personalized Probiotics

Personalized probiotics represent a transformative advancement in microbiome therapeutics, shifting from generalized formulations to precision interventions tailored to individual gut microbiota profiles. Through the integration of multi-omics technologies—including metagenomic sequencing, metabolomics, and bioinformatics—this approach enables the comprehensive analysis of an individual’s gut microbial composition, functional pathways, and metabolic activity [157].

Beyond therapeutic applications, personalized probiotics show promise for preventive health, using predictive analytics to identify at-risk individuals and pre-emptively address microbial imbalances [158]. This paradigm aligns with the broader precision medicine movement, emphasizing dynamic, data-informed adjustments to formulations as an individual’s microbiome evolves with their age, diet, and environmental exposures [159]. However, challenges remain regarding standardizing microbial characterization methods, validating strain synergies, and ensuring scalability. Nevertheless, as research continues to unravel the complex interplay between the microbiota and human physiology, personalized probiotics are positioned to redefine microbiome management, offering bespoke solutions that bridge microbial ecology with clinical outcomes. Despite their potential, personalized probiotics face challenges in the areas of R&D, process standardization, and cost acceptability, among other obstacles. Overcoming these hurdles to make personalized probiotics widely accessible remains a significant challenge for researchers.

### 6.2. Regulatory and Standardization Challenges

1. Security assessment

Objectively speaking, existing research on probiotic encapsulation materials has seldom addressed the validation phase for commercialization, such as impacts on sensory characteristics or bio-toxicity assessments, which are critical for safety evaluation. Overall, the long-term biological safety of synthetic materials still requires further verification. Nanoparticles, in particular, may accumulate within the body—especially in organs such as the liver—necessitating comprehensive toxicological studies to determine their safety thresholds [160]. Furthermore, regulatory frameworks for genetically modified microorganisms remain underdeveloped, with an urgent need to establish evaluation standards for their genetic stability and horizontal gene transfer risks to ensure the safe application of these technologies.

2. Labels and efficacy claims

At present, there is no unified standard for health claims regarding probiotic products, with regulatory requirements for probiotics varying significantly across different countries and regions. For instance, some regions mandate at least one randomized controlled trial (RCT) as evidence to support efficacy claims, while others adopt more flexible certification methods [161]. Moving forward, international coordination should be promoted to establish a standardized labeling system based on strain specificity, dosage, and clinical endpoints. This can be expected to provide consumers with a clearer understanding of a product’s actual efficacy, rather than allowing misleading probiotic labels to deceive consumers into purchasing products that lack proven effects.

3. Production quality control

To ensure consistency between different batches of products, strict quality control indicators must be established, including key parameters such as encapsulation efficiency, survival rate, and release kinetics [35]. Through the adoption of advanced technological means such as blockchain technology, transparent management of the entire chain from raw material procurement to finished product delivery can be achieved, thereby enhancing consumer trust. This can be expected to not only help in improving product quality but may also promote the healthy development of the entire industry.

## 7. Conclusions

Encapsulation technology plays a crucial role in enhancing the viability, stability, and targeted delivery of probiotics. This review systematically discussed key encapsulation techniques—including physical, chemical, and advanced multilayer approaches—highlighting their effectiveness in protecting probiotics from harsh environmental conditions such as gastric acidity, oxygen exposure, and storage instability. Furthermore, encapsulation not only improves the survival rate of probiotics during processing and gastrointestinal transit but also enables their controlled release and enhanced interaction with the gut microbiota, thus maximizing their health-promoting effects. Despite significant advancements several challenges remain, including the high costs of encapsulation materials and equipment, scalability issues, and strain-specific variability regarding survival and functionality. Future research should focus on developing cost-effective, scalable encapsulation strategies while integrating innovative materials and precision-targeted delivery systems. Additionally, personalized probiotic formulations based on individual gut microbiome profiles and regulatory standardization efforts are expected to be critical in advancing probiotic-based functional foods and therapeutic applications.

## Figures and Tables

**Table 1:** Advantages and disadvantages of different probiotic encapsulation methods.

Method	Formation Mechanism	Advantages	Disadvantages	Ref.
Spray Drying	The probiotic suspension is dispersed and then forms dry powder particles in the hot air flow.	1. Energy-efficient. 2. Low cost. 3. Suitable for large-scale production.	1. High temperatures damage probiotics. 2. Porous particle structure. 3. Broad size distribution.	[20,21]
Freeze Drying	Moisture removal via ice sublimation under vacuum after low-temperature freezing.	1. Maximizes viability retention. 2. Ideal for heat-sensitive strains.	1. Long production cycle. 2. High energy consumption. 3. Risk of ice crystal damage.	[22,23]
Coacervation	Electrostatic deposition of oppositely charged biopolymers on cell surfaces.	1. High encapsulation efficiency. 2. Mild conditions.	1. Complex parameter control. 2. Often requires additional cross-linking steps.	[24]
Endogenous Emulsion	Construction of multiple functional polymer shells via layer-by-layer assembly.	1. Outer layer provides acid/stress resistance. 2. Inner layer offers nutrients/buffering.	1. Tedious multi-step process. 2. Interlayer instability. 3. Difficult industrial scalability.	[25,26]
Multi-Layer Encapsulation	Sequential coating with multiple materials (e.g., alginate and chitosan).	1. Enhanced protection against harsh environments.	1. Complex fabrication. 2. Higher cost.	[27,28]
Cell-Mediated Nanocoatings	In situ self-assembly or coordination driven by cell surface templates or secretions.	1. No mass transfer limitation for nutrients. 2. Negligible impact on product’s sensory texture.	1. Potential toxicity of specific reaction conditions to sensitive strains.	[29]
Dual-Core Co-Encapsulation	Co-encapsulation of probiotics and prebiotics/bioactives as independent or mixed cores within a single matrix.	Facilitates synergistic (synbiotic) effects.	1. Complex internal structure. 2. Difficult synchronization of release kinetics. 3. Limited loading capacity.	[30]

**Table 2:** Encapsulation efficiency and survival rates of probiotics using various encapsulation methods and materials.

Method	Probiotics	Coating Material	Encapsulation Efficiency (%)	Survival Rate (%)		Ref.
				in SGF	in SIF	
Spray drying	*L. acidophilus* LA-5	Modified starch	88.55	85.99	-	[133]
	*L. acidophilus* La-5	Inulin	~66	-	-	[134]
	*L. acidophilus*	Gum arabic, β-cyclodextrin	93.51	84.05	96.04	[135]
	*B. bifidum*		82.02	90.10	87.94	
Freeze drying	*L. rhamnosus* GG	Whey proteins, galactooligosaccharides, fructooligosaccharides	95.08	58.88	46.77	[136]
	*Limosilactobacillus fermentum*	Whey protein, gum arabic	94.69	-	-	[137]
Coacervation	*L. Plantarum* WCFS1	Whey protein isolate, gum arabic	-	93.33	90.50	[138]
	*L. plantarum*	Gelatin, sodium carboxymethyl cellulose	87.27	55.41	-	[139]
Endogenous emulsion	*Lacticaseibacillus paracasei* ProSci-92	SA, soy protein	92.17	84.17	80.20	[40]
Multi-layer encapsulation	*L. rhamnosus* 6133	Hyaluronan, gelatin	92	-	-	[27]
	*L. paracasei* ML33, *L. paracasei* ML82	SA, maize starch	98.71	>94	>94	[140]
Cell-mediated nanocoatings	*L. rhamnosus*	Polydopamine	99	62	73	[29]
	*L. acidophilus*	Eggshell membrane hydrolysates, coffee melanoidins	93.8	79	-	[141]
Dual-core co-encapsulation	*L. plantarum* NCDC 414	Inulin, dextran, maltodextrin	99.21	-	-	[142]
	*Bacillus coagulans*	Gellan/κ-carrageenan/chitosan tri-composite hydrogel	89.56	-	-	[143]

## Data Availability

No new data were created or analyzed in this study. Data sharing is not applicable to this article.
